# Prediction of drug–target binding affinity using similarity-based convolutional neural network

**DOI:** 10.1038/s41598-021-83679-y

**Published:** 2021-02-24

**Authors:** Jooyong Shim, Zhen-Yu Hong, Insuk Sohn, Changha Hwang

**Affiliations:** 1 Department of Statistics, Institute of Statistical Information, Inje University, Gimhae, Gyeongsangnamdo South Korea; 2 Arontier, Seoul, 06735 South Korea; 3 Department of Applied Statistics, Dankook University, Yongin, Gyeonggido 16890 South Korea

**Keywords:** Computational biology and bioinformatics, Drug discovery

## Abstract

Identifying novel drug–target interactions (DTIs) plays an important role in drug discovery. Most of the computational methods developed for predicting DTIs use binary classification, whose goal is to determine whether or not a drug–target (DT) pair interacts. However, it is more meaningful but also more challenging to predict the binding affinity that describes the strength of the interaction between a DT pair. If the binding affinity is not sufficiently large, such drug may not be useful. Therefore, the methods for predicting DT binding affinities are very valuable. The increase in novel public affinity data available in the DT-related databases enables advanced deep learning techniques to be used to predict binding affinities. In this paper, we propose a similarity-based model that applies 2-dimensional (2D) convolutional neural network (CNN) to the outer products between column vectors of two similarity matrices for the drugs and targets to predict DT binding affinities. To our best knowledge, this is the first application of 2D CNN in similarity-based DT binding affinity prediction. The validation results on multiple public datasets show that the proposed model is an effective approach for DT binding affinity prediction and can be quite helpful in drug development process.

## Introduction

Drug-target interactions (DTIs) characterize the binding of drug compounds to the protein targets. Drug screening and repurposing are two main applications associated with DTIs^1^. Therefore, identifying novel DTIs is a crucial step in drug discovery process. Most methods developed to predict DTIs have focused on binary classification^2–9^, which neglects DT binding affinity (DTA) reflecting how strongly the drug binds to its target. In fact, DTI depends on several factors such as the concentrations of the two molecules and their intermolecular interactions. Thus, DTA prediction problem is regarded as a regression task where the input is a pair of DT representations and the output is a continuous value reflecting DTA. Binding affinity is generally expressed in terms of dissociation constant ($K_{d}$), inhibition constant ($K_{i}$) or the half maximal inhibitory concentration ($IC_{50}$). The $IC_{50}$ value depends on the concentration of the target and ligand^10^ and low $IC_{50}$ values mean high binding affinity. Similarly, low $K_{i}$ values mean high binding affinity. In general, $K_{d}$ and $K_{i}$ values are expressed in terms of $pK_{d}$ and $pK_{i}$ respectively, which stand for the negative logarithm of $K_{d}$ and $K_{i}$.

DTA prediction has been the focus of DT scoring, which is used to estimate the binding strength and predict the effective DT binding after virtual screening and docking campaigns^11^. Machine learning (ML) regression methods such as the random forest (RF) and support vector regression (SVR) have been used as a successful alternative to scoring functions that depend on multiple parameters^12–14^. However, Gabel et al.^15^ demonstrated that the ML scoring functions failed in virtual screening and docking tests because they were overtrained on descriptors that do not depict DTIs but interaction-independent counts. Since then, deep learning has begun to become a popular architecture due to the increase in data and high performance computing machines.

Inspired by the remarkable advances in image processing^16–18^ and speech recognition^19–21^, deep learning methods were actively used in many other research areas, including genomics studies^22,23^ and quantitative-structure activity relationship (QSAR) studies^24^. Deep learning methods were also used to predict DTI employing deep neural networks (DNNs)^25–27^, convolutional neural networks (CNNs)^28,29^, stacked autoencoders^30^ and deep belief networks^31^.

Machine learning approaches were recently applied to predict continuous DTA values. Pahikkala et al.^32^ introduced the Kronecker regularized least squares (KronRLS) model which utilizes only compound similarity-based representations of the drugs and Smith–Waterman similarity^33^ representation of the targets. KronRLS model utilizes conventional machine learning technique. Öztürk et al.^34^ proposed DeepDTA model with two 1-dimensional (1D) CNN blocks to learn from compound SMILES strings and protein sequences. The simple concatenation of two feature representations from two CNN blocks is fed into one or more fully connected (FC) layers followed by the output layer. Zhao et al.^35^ proposed a semi-supervised generative adversarial networks^36^ (GANs)-based method to predict binding affinity, called GANsDTA for short. GANsDTA model can learn proteins and drugs features of both labeled and unlabeled data by utilizing two GANs. Abbas et al.^37^ proposed DeepCDA model which utilizes a combination of CNN and long-short-term memory (LSTM) to get a better representation of protein and compound. DeepCDA encodes the binding strength by utilizing a two-sided attention mechanism and tries to improve the generalization ability by utilizing a domain adaptation technique.

**Figure 1:**
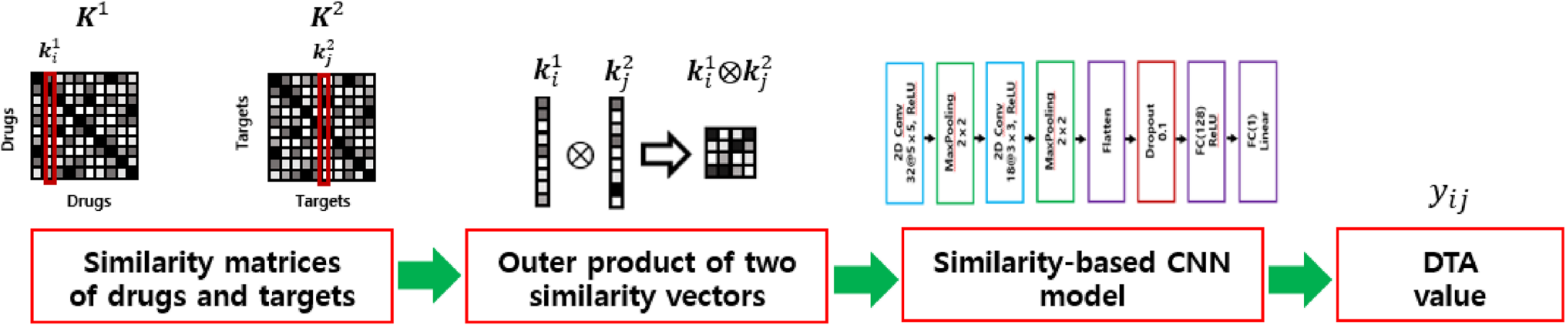
A workflow of the proposed SimCNN-DTA for predicting DTA values. Given an outer product of drug similarity vector and target similarity vector as input, 2D CNN is used to learn features. The architecture of CNN consists of 2 convolutional layers, 2 max-pooling layers, 1 flatten layer, 1 dropout layer and 2 fully connected (FC) layers.

In this paper, we propose a similarity-based model to predict DTA values using 2D CNN approach on the outer product of column vectors of Tanimoto similarity matrix and Smith–Waterman similarity matrix for the drugs and targets, respectively^32,38^. This model is called SimCNN-DTA for short. Figure 1 explains the entire workflow of the proposed SimCNN-DTA for the prediction of DTA values. Our method consists of two stages. First, Tanimoto similarity matrix of the drugs and Smith–Waterman similarity matrix for the targets are calculated and then the outer products between column vectors of these two matrices are calculated. Second, a 2D CNN model is adopted to extract deep features from the outer products and to predict DTA values.

Our SimCNN-DTA is verified via mean squared error (MSE), concordance index (CI), modified squared correlation coefficient $r_{m}^{2}$ and the area under precision recall (AUPR) score on the Davis kinase binding affinity dataset^38^ and the large-scale kinase inhibitors bioactivity (KIBA) dataset^38,40^. Our SimCNN-DTA is also verified via a case study using actual USFDA-approved drugs targeting a specific protein, epidermal growth factor receptor (EGFR). We also compare our model with the KronRLS^33^ and deepDTA^34^ algorithm. Our research results might be helpful for users to predict DTA values.

## Results and discussion

### Experimental datasets

In the paper we use the Davis dataset and the KIBA dataset for the evaluation of DTA predictions^32,34,39^. Both datasets are large scale biochemical selectivity assays of the kinase inhibitors. The Davis dataset includes binding affinities observed for all pairs of 68 drugs and 442 targets, measured by $K_{d}$ value. On the other hand, the KIBA dataset integrates kinase inhibitor bioactivities from different sources such as $K_{d}$, $K_{i}$, and $IC_{50}$ into KIBA score which represents the binding affinity. The lower KIBA score indicates a higher binding affinity between a DT pair. The KIBA dataset originally comprises 52,498 drugs and 467 targets, including 246,088 observations. However, it results in a dataset of 2,116 drugs and 229 targets with a density of 24.4%, by removing all drugs and targets with less than 10 interactions. Table 1 describes these two datasets in the forms they are actually used in the experiment. Pahikkala et al.^32^ directly used the $K_{d}$ values in the Davis dataset as the binding affinity values. However, we here use the values transformed into logspace, $pK_{d}$, similar to Öztürk et al.^34^ and He et al.^39^, explained as follows:

1 $$ pK_{d} = - {\text{log}}_{10} \left( {\frac{{K_{d} }}{{1e^{9} }}} \right) $$

**Table 1:** The statistics of two datasets.

Dataset	Drugs	Targets	Interactions	Density (%)
Davis	68	442	30,056	100
KIBA	2111	229	118,254	24.4

**Figure 2:**
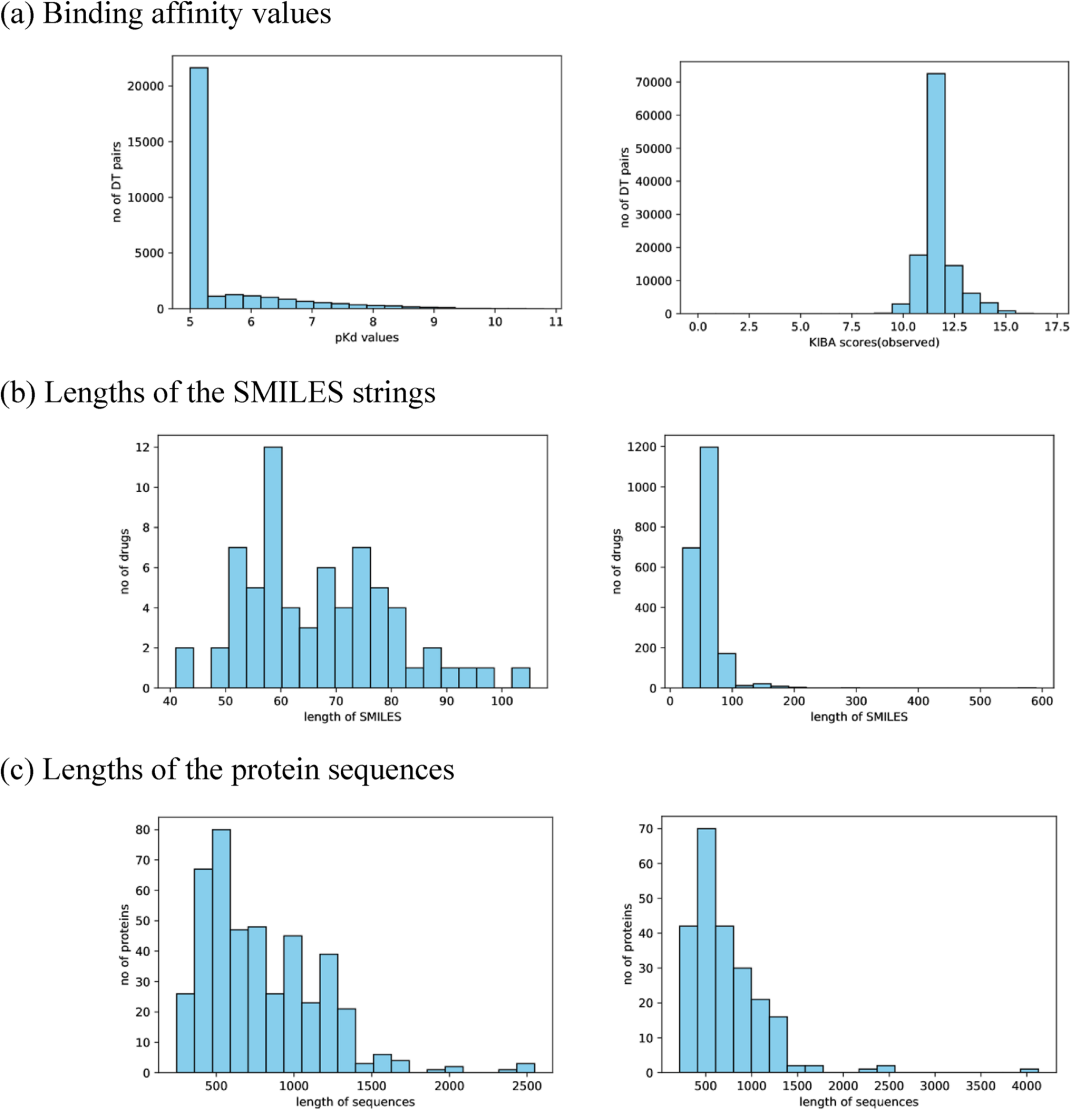
Summary of the Davis dataset (left panel) and KIBA (right panel) dataset. (**a**) Distribution of binding affinity values. (**b**) Distribution of the lengths of the SMILES strings. (**c**) Distribution of the lengths of the protein sequences.

The left panel of Fig. 2a shows the distribution of the binding affinity values in $pK_{d}$ form. The higher the $pK_{d}$ value, the higher the binding affinity. We observe a strong peak at $pK_{d}$ value 5 for the Davis dataset, since weak binding affinities are often reported as $K_{d} \ge 10,000$ or $pK_{d} \le 5$. The right panel of Fig. 2a shows the distribution of the KIBA scores. In the KIBA dataset, the lower the KIBA score, the higher the binding affinity. Tang et al.^39^ suggests a threshold of KIBA score $\le 3.0$ to binarize the dataset. Similar to Öztürk et al.^34^ and He et al.^38^, we transform the KIBA scores as follows: (1) for each KIBA score, its negative is taken, (2) the minimum value among the negatives is chosen and (3) the absolute value of the minimum is added to all scores. Thus, the original KIBA threshold of 3.0 becomes 12.1 in the transformed dataset.

Figure 2b shows the distribution of the lengths of the SMILES strings of the drugs in the Davis (left) and KIBA (right) datasets. For the drugs of the Davis dataset, the maximum of SMILES lengths is 103 and the average is 64. For the drugs of KIBA dataset, the maximum of SMILES length is 590 and the average is 58. For the Davis dataset, the SMILES strings were extracted from the PubChem compound database utilizing their PubChem CIDs^40^. For the KIBA dataset, the ChEMBL IDs were first converted into PubChem CIDs and then the SMILES strings were extract utilizing the corresponding CIDs.

Figure 2c shows the distribution of the lengths of the sequences of the proteins in the Davis (left) and KIBA (right) datasets. For the proteins of the Davis dataset, the maximum of sequence lengths is 2549 and the average is 788. For the proteins of the KIBA dataset, the maximum of sequence lengths is 4128 and the average is 728. The protein sequences of the Davis dataset were extracted from the UniProt protein database utilizing gene names/RefSeq accession numbers^41^. The protein sequences of the KIBA dataset were extracted utilizing the UniProt IDs.

We also use actual USFDA-approved drugs targeting a specific protein EGFR. This protein is chosen to be the target since this is known as one of the famous genes associated with many cancer types. We will predict the binding affinities between the EGFR and the 1,018 drugs, of which 11 drugs are known to be EGFR targeting drugs.

### Input and output representations

In our SimCNN-DTA, drug-drug and target-target similarity matrices for the Davis, KIBA and EGFR datasets are used. These matrices are denoted ${\varvec{K}}^{1}$ and ${\varvec{K}}^{2}$, respectively. The input of SimCNN-DTA for each DT pair is the outer product ${\varvec{k}}_{i}^{1} \otimes {\varvec{k}}_{j}^{2}$ of ${\varvec{k}}_{i}^{1}$ and ${\varvec{k}}_{j}^{2}$, where ${\varvec{k}}_{i}^{1} = \left( {k_{i1}^{1} ,k_{i2}^{1} , \ldots ,k_{im}^{1} } \right)^{t}$ is the $i$th column of similarity matrix ${\varvec{K}}^{1}$, ${\varvec{k}}_{j}^{2} = \left( {k_{j1}^{2} ,k_{j2}^{2} , \ldots ,k_{jn}^{2} } \right)^{t}$ is the $j$th column of similarity matrix ${\varvec{K}}^{2}$ and $\otimes$ stands for the outer product. The superscript $t$ represents the transpose of vector. The outer product ${\varvec{k}}_{i}^{1} \otimes {\varvec{k}}_{j}^{2}$ is actually defined as follows:

2 $$ {\varvec{k}}_{i}^{1} \otimes {\varvec{k}}_{j}^{2} = \left( {\begin{array}{*{20}c} {\begin{array}{*{20}c} {k_{i1}^{1} k_{j1}^{2} } & {k_{i1}^{1} k_{j2}^{2} } \\ {k_{i2}^{1} k_{j1}^{2} } & {k_{i2}^{1} k_{j2}^{2} } \\ \end{array} } & {\begin{array}{*{20}c} \cdots & {k_{i1}^{1} k_{jn}^{2} } \\ \cdots & {k_{i2}^{1} k_{jn}^{2} } \\ \end{array} } \\ {\begin{array}{*{20}c} \vdots & \vdots \\ {k_{im}^{1} k_{j1}^{2} } & {k_{im}^{1} k_{j2}^{2} } \\ \end{array} } & {\begin{array}{*{20}c} \ddots & \vdots \\ \cdots & {k_{im}^{1} k_{jn}^{2} } \\ \end{array} } \\ \end{array} } \right) $$

This outer product produces two sets of information: the bimodal interactions and the raw unimodal representations of the individual modalities, since $k_{ii}^{1} = 1$ and $k_{jj}^{2} = 1$. Therefore, ${\varvec{k}}_{i}^{1} \otimes {\varvec{k}}_{j}^{2}$ contains the full combinations of information of ${\varvec{k}}_{i}^{1}$ and ${\varvec{k}}_{j}^{2}$. This implies that ${\varvec{k}}_{i}^{1} \otimes {\varvec{k}}_{j}^{2}$ could be the better input than the simple concatenation of ${\varvec{k}}_{i}^{1}$ and ${\varvec{k}}_{j}^{2}$ for prediction of the binding affinity of $i$th drug and $j$th protein.

The drug-drug similarity is computed using the Tanimoto coefficient $T$, which is the most popular similarity measure for comparing chemical structures represented by means of fingerprints. In the paper, we use the topological fingerprint of the RDKit. On the other hand, the target-target similarity is computed based on the protein sequences, using the normalized Smith-Waterman score as explained in the Eq. (3). This guarantees to calculate the optimal score between any two protein sequences.

3 $$ SW_{ij}^{st} = \frac{{SW_{ij} }}{{\sqrt {SW_{ii } SW_{jj} } }} $$

The output for each DT pair is the corresponding binding affinity value $y_{ij}$.

### Performance evaluation metrics

Since KronRLS and DeepDTA are typical computational non-structure-based methods devised for predicting DTAs, we consider them the baseline methods. In the paper, we compare the performance of KronRLS, DeepDTA and SimCNN-DTA, using the aforementioned datasets for evaluation. We perform threefold and fivefold cross validation (CV) experiments on the Davis and KIBA datasets. To ensure that no target is used only for training or only for testing, we build the folds in a way such that every target has an observation in at least two and four folds, respectively. We use four metrics such as MSE, CI, $r_{m}^{2}$ and AUPR for the evaluation of the performance in these regression-based models.

The MSE is a commonly used metric for the error in continuous prediction. Since we work on a regression task, we use the MSE as the metric commonly used for the error in continuous prediction, in which $y_{i}$ is the actual output and $\hat{y}_{i}$ corresponds to the prediction. $n$ indicates the number of samples.

4 $$ MSE = \frac{1}{n}\mathop \sum \limits_{i = 1}^{n} \left( {y_{i} - \hat{y}_{i} } \right)^{2} $$

As suggested in Pahikkala et al.^32^, the CI can be used as an evaluation metric for the prediction accuracy. The intuition behind the CI is as follows: the CI over a set of paired data is the probability that the predictions for two randomly drawn DT pairs with different label values are in the correct order, meaning that the prediction $\hat{y}_{i}$ for the larger affinity $y_{i}$ is larger than the prediction $\hat{y}_{j}$ for the smaller affinity value $y_{j}$:

5 $$ CI = \frac{1}{Z}\mathop \sum \limits_{{y_{i} > y_{j} }} h\left( {\hat{y}_{i} - \hat{y}_{j} } \right) $$

where $Z$ is a normalization constant and $h\left( x \right)$ is the step function^32^:

6 $$ h\left( x \right) = \left\{ {\begin{array}{*{20}c} {\begin{array}{*{20}c} {1,} & { x > 0} \\ \end{array} } \\ {\begin{array}{*{20}c} {0.5,} & {x = 0} \\ \end{array} } \\ {\begin{array}{*{20}c} {0,} & { x < 0} \\ \end{array} } \\ \end{array} } \right. $$

The CI ranges between 0.5 and 1.0, where 0.5 corresponds to a random predictor and 1.0 corresponds to perfect prediction accuracy.

For better predictive potential of the model, a modified squared correlation coefficient $r_{m}^{2}$ was introduced by Roy and Roy^43^ as follows:

7 $$ r_{m}^{2} = r^{2} \left( {1 - \sqrt {r^{2} - r_{0}^{2} } } \right) $$

where $r^{2}$ and $r_{0}^{2}$ are the squared correlation coefficients with and without intercept, respectively. It is known that the model with $r_{m}^{2} > 0.5$ for the test dataset is determined as an acceptable model.

The AUPR score is widely utilized by many studies on binary prediction. In order to compute AUPR, we convert the Davis and KIBA datasets into their binary forms through selecting appropriate binding affinity thresholds. For Davis dataset we use $pK_{d}$ value of 7.0 as threshold as in Pahikkala et al.^32^. For KIBA dataset we use KIBA value of 12.1 as the suggested threshold^32,34,36^.

### Learning and evaluation

We now illustrate the prediction performance of the proposed SimCNN-DTA for predicting DTA. We evaluate the performance of the proposed model on two benchmark datasets similar to Öztürk et al.^34^ and He et al.^39^. As mentioned before, we perform nested threefold and fivefold CVs on the Davis and KIBA datasets. Table 2 illustrates the performance results for five models via nested threefold and fivefold CVs on the Davis dataset, respectively. Table 3 illustrates the performance results for five models via nested threefold and fivefold CVs on the KIBA dataset, respectively. Boldfaced values indicate best performance result. Standard errors are given in parenthesis.

**Table 2:** Comparison of five prediction models via threefold and fivefold cross validations on the Davis dataset.

Model	Threefold CV				Fivefold CV			
	MSE	CI	$r_{m}^{2}$	AUPR	MSE	CI	$r_{m}^{2}$	AUPR
KronRLS	0.3614*** (0.0023)	**0.8669** (0.0027)	0.4751*** (0.0007)	0.5902*** (0.0019)	0.3923**** (0.0073)	**0.8767** (0.0024)	0.5120*** (0.0043)	0.6080*** (0.0074)
DeepDTA	0.4126*** (0.0106)	0.8313**** (0.0019)	0.4923** (0.0072)	0.5823*** (0.0020)	0.3856** (0.0196)	0.8460*** (0.0026)	0.5331* (0.0213)	0.6059** (0.0121)
GANsDTA	0.5018**** (0.0044)	0.7958**** (0.0030)	0.3620*** (0.0160)	0.4927**** (0.0034)	0.4895**** (0.0071)	0.7984**** (0.0026)	0.3704**** (0.0110)	0.5109**** (0.0052)
DeepCDA	0.4590** (0.0317)	0.8396*** (0.0020)	0.5029** (0.0056)	0.5797*** (0.0060)	0.3884**** (0.0124)	0.8528** (0.0044)	0.5512* (0.0164)	0.5983*** (0.0054)
SimCNN-DTA	**0.3190** (0.0041)	0.8501*** (0.0011)	**0.5655** (0.0077)	**0.6511** (0.0077)	**0.3059** (0.0022)	0.8552** (0.0027)	**0.5952** (0.0138)	**0.6572** (0.0076)

**Table 3:** Comparison of five prediction models via threefold and fivefold cross validations on the KIBA dataset.

Model	Threefold CV				Fivefold CV			
	MSE	CI	$r_{m}^{2}$	AUPR	MSE	CI	$r_{m}^{2}$	AUPR
KronRLS	0.2806* (0.0021)	0.7948**** (0.0004)	0.5438** (0.0020)	0.7001^+^ (0.0014)	0.2616^+^ (0.0015)	0.8060*** (0.0007)	0.5667* (0.0010)	0.7129** (0.0007)
DeepDTA	0.5285* (0.0723)	0.7666**** (0.0019)	0.4746**** (0.0054)	0.6304**** (0.0012)	0.4715* (0.0766)	0.7824**** (0.0025)	0.5209** (0.0112)	0.6540**** (0.0034)
GANsDTA	0.3816**** (0.0045)	0.7619**** (0.0012)	0.4449**** (0.0047)	0.6003**** (0.0014)	0.3878**** (0.0037)	0.7592**** (0.0026)	0.4391**** (0.0043)	0.5972**** (0.0042)
DeepCDA	0.4980^+^ (0.1177)	0.7773**** (0.0004)	0.5051**** (0.0034)	0.6259**** (0.0040)	0.6763* (0.1279)	0.7789**** (0.0021)	0.5165** (0.0072)	0.6311*** (0.0081)
SimCNN-DTA	**0.2740** (0.0019)	**0.8141** (0.0011)	**0.5618** (0.0026)	**0.7047** (0.0019)	**0.2576** (0.0018)	**0.8216** (0.0011)	**0.5734** (0.0034)	**0.7213** (0.0018)

To statistically evaluate the significant improvement of our method, we utilize the one sided $t$-test. We basically compare the model with the best performance result to other models. Thus, the null hypotheses associated with Table 2 are given as $H_{0} :MSE\left( {\text{other model}} \right) \le MSE\left( {{\text{SimCNN}} - {\text{DTA}}} \right)$, $H_{0} :CI\left( {{\text{KronRLS}}} \right) \le CI\left( {\text{other model}} \right)$, $H_{0} :r_{m}^{2} \left( {{\text{SimCNN}} - {\text{DTA}}} \right) \le r_{m}^{2} \left( {\text{other model}} \right)$, $H_{0} :AUPR\left( {{\text{SimCNN}} - {\text{DTA}}} \right) \le AUPR\left( {\text{other model}} \right)$. The null hypotheses associated with Table 3 are the same as the null hypotheses associated with Table 2 except the second hypothesis, $H_{0} :CI\left( {{\text{SimCNN}} - {\text{DTA}}} \right) \le CI\left( {\text{other model}} \right)$. The relevant $p$-values less than 0.1 are given + , $p$-values less than 0.05 are given one asterisk, $p$-values less than 0.01 are given two asterisks, $p$-values less than 0.001 are given three asterisks, and $p$-values less than 0.0001 are given four asterisks.

For all evaluation metrics, the value for the best-performing model is highlighted in bold font. As seen from Table 2, our approach significantly outperforms the other models for all three metrics except CI for Davis dataset. As seen from Table 3, our approach significantly outperforms the other models for all four metrics for KIBA dataset. As seen from both tables, the similarity-based models, KronRLS and SimCNN-DTA tend to perform better than deepDTA, GANsDTA and deepCDA learned from drug SMILES strings and protein sequences. SimCNN-DTA overall performs better than KronRLS. SimCNN-DTA is most acceptable model, since it obtains significantly bigger $r_{m}^{2} > 0.5$ than the other models for threefold and fivefold CVs on the Davis and KIBA datasets. It is noteworthy that SimCNN-DTA only use nonmissing DTA values for training, whereas KronRLS uses both missing and nonmissing DTA values. The missing affinity of a drug is imputed with the weighted average of nonmissing affinities, where the weights are the normalized Smith–Waterman similarities between the protein corresponding to the missing affinity and proteins corresponding to the nonmissing affinities.

**Figure 3:**
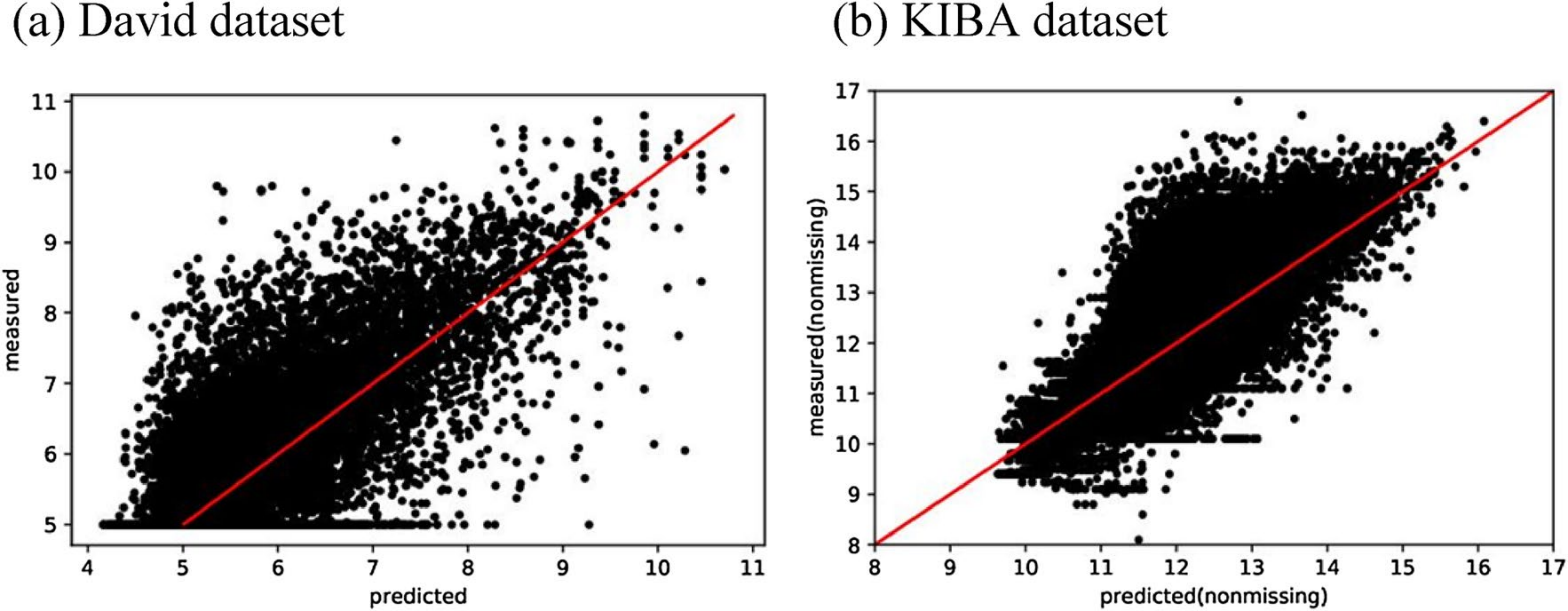
Scatter plots of predictions of SimCNN-DTA versus measured binding affinity values for Davis ($pK_{d}$) and KIBA (KIBA score) datasets.

Figure 3 shows the scatter plots of the predicted DTA values of SimCNN-DTA versus the measured DTA values on Davis and KIBA datasets. A perfect regression model is expected to be a $\hat{y} = y$ line where predictions ($\hat{y}$) are equal to the measured ($y$) values. For better illustration of KIBA dataset we set the range of scatter plot to $\left[ {8,{ }\infty } \right]$, since only 13 out of 118,254 nonmissing values in KIBA dataset have scores less than 8. We notice that the density is high around the $\hat{y} = y$ line particularly for KIBA dataset.

We now illustrate a case study using actual USFDA-approved drugs targeting the specific protein EGFR. We predict the affinities between EGFR and the 1,018 drugs, of which 11 drugs are known to be EGFR targeting drugs. We train KronRLS, DeepDTA and SimCNN-DTA using the Davis dataset as training dataset. The predicted affinities between 11 drugs and EGFR are sorted in descending order and summarized in Table 4. The results indicate that SimCNN-DTA successfully identifies known EGFR targeting drugs and other chemical compounds for which no association with EGFR was reported.

**Table 4:** Compound ranking based on the predicted affinities of SimCNN-DTA when the target is EGFR.

PubChem ID	KronRLS ranking	DeepDTA ranking	GANsDTA ranking	DeepCDA ranking	SimCNN-DTA ranking
DB11963	2	459	393	425	1
DB08916	1	306	33	306	2
DB00317	3	351	148	445	3
DB11828	4	461	927	472	4
DB00530	5	664	61	474	5
DB01259	7	285	516	506	6
DB05294	6	455	423	492	7
DB12010	9	122	162	68	8
DB12267	13	517	863	225	10
DB09330	18	370	966	453	30
DB00281	667	933	337	875	90

## Conclusion

In this paper we proposed the novel method SimCNN-DTA for the problem of predicting continuous DTAs. As discussed above, continuous values provide more information about the actual strength of DT binding. To the best of our knowledge, SimCNN-DTA is the first nonlinear method that applies 2D CNN approach to the outer products between column vectors of Tanimoto similarity matrix of the drugs and column vectors of Smith–Waterman similarity matrix of the targets for continuous DTA prediction.

Experimental results show that SimCNN-DTA outperforms other existing methods-KronRLS and DeepDTA in prediction performance on the Davis and KIBA datasets. Moreover, case study of finding drug candidates targeting EGFR shows that SimCNN-DTA successfully includes all existing EGFR drugs as 100 top-ranked candidates among 1,018 candidates.

SimCNN-DTA can be further improved by adjusting the architecture of CNN according to the data structure. To the best of our knowledge, we believe that SimCNN-DTA is an effective approach for DTA prediction and can be quite helpful in drug development process.

## Material and methods

### Baseline models

For the baseline models, one similarity-based model and three deep learning-based models are taken into consideration. The similarity-based model is KronRLS^32^, which aims to minimize a standard squared error loss function with a specific regularization term. The regularization term is given as a norm of the prediction function, which is related with a kernel function. Deep learning models such as DeepDTA^34^, GANsDTA^35^ and DeepCDA^37^ are the state-of-the-art methods in predicting DTAs, which are 1D CNN-based prediction models using integer/label encoded SMILES strings and protein sequences. DeepDTA^34^ consists of two separate CNN blocks, each aiming to learn representations from compound SMILES strings and protein sequences, composed of three consecutive 1D convolutional layers followed by a max-pooling layer. At the end of CNN blocks, two outputs are concatenated into a vector, which is fed into three FC layers. Then this model predicts affinity scores from the model. GANsDTA^35^ is the 1D CNN-based prediction model using the combined features extracted from integer/label encoded SMILES strings and protein sequences by GANs as the inputs, where 1D CNN consists of three consecutive 1D convolutional layers with kernels of size 4. DeepCDA^37^ consists of three steps. In the first step, a combination of CNN and LSTM is proposed to obtain a better representation of protein and compound. Also, a two-sided attention mechanism is proposed to encode the interaction strength between each compound substructure and protein substructure pair. In the second step, a feature encoder network is learned to improve the generalizability of the model by utilizing an adversarial domain adaptation technique. In the third step, the learned test encoder network is applied to new compound–protein pairs to predict their binding affinity.

### SimCNN

The proposed SimCNN model is the 2D CNN-based prediction model that uses the outer product of drug similarity vector and protein similarity vector as input. For the input of our SimCNN model, we first calculate $n_{d} \times n_{d}$ drug-drug similarity matrix ${\varvec{K}}^{1}$ based on Tanimoto coefficient and $n_{t} \times n_{t}$ target-target similarity matrix ${\varvec{K}}^{2}$ based on the normalized Smith-Waterman score for datasets. The topological fingerprint of the RDKit is used to calculate Tanimoto coefficient. The normalized Smith-Waterman score guarantees to calculate the optimal score between any two target sequences. Here, $n_{d}$ and $n_{p}$ are the number of drugs and targets in training dataset, respectively. Then, we calculate the outer product ${\varvec{k}}_{i}^{1} \otimes {\varvec{k}}_{j}^{2}$ of $n_{d} \times 1$ drug similarity vector ${\varvec{k}}_{i}^{1}$ and $n_{p} \times 1$ protein similarity vector ${\varvec{k}}_{j}^{2}$ for all DT pairs, where ${\varvec{k}}_{i}^{1}$ and ${\varvec{k}}_{j}^{2}$ are the $i$th column and the $j$th column of similarity matrices ${\varvec{K}}^{1}$ and ${\varvec{K}}^{2}$, respectively. That is to say, ${\varvec{k}}_{i}^{1}$ consists of Tanimoto similarities between the $i$th drug and others including itself, and ${\varvec{k}}_{j}^{2}$ consists of normalized Smith-Waterman similarities between the $j$th target and others including itself.

**Figure 4:**
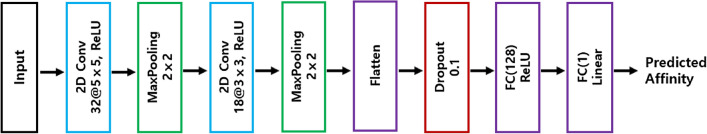
The architecture of SimCNN-DTA.

The parameters associated with SimCNN are obtained using outer products as inputs and affinities as outputs. As seen in Fig. 4, our SimCNN-DTA consists of two 2D convolutional layers, each followed by a max-pooling, 1 flatten layer, FC(128) and FC(1) layers. The numbers in parentheses indicate the number of nodes. FC(128) and FC(1) layers use the rectified linear unit (ReLU) and linear functions, respectively. We add dropout layer with rate 0.1 between the flatten layer and FC(128) layer to eliminate overfitting. The filter numbers for each convolutional layer are 32 and 18, respectively. We use filters of kernel size of 5 × 5, 3 × 3 respectively for convolutional layers. We use max-pooling of size 2, stride 1. We set the batch size and number of epochs with the 32 and 20 respectively for a learning algorithm. We use Adam optimizer with learning rate 0.001.
